# Validation of Commercial Pasteurization and Shelf-Life Evaluation of Finely Minced Cooked Chicken Sausages in Wide-Diameter Polyamide Casings Using *p* Values

**DOI:** 10.3390/foods15152710

**Published:** 2026-07-31

**Authors:** Mladen Rašeta, Mirjana Lukić, Caba Siladji, Lazar Milojević, Damjan Gavrilović, Dunja Videnović, Jelena Jovanović

**Affiliations:** Institute of Meat Hygiene and Technology, Kaćanskog 13, 11040 Belgrade, Serbia; mirjana.lukic@inmes.rs (M.L.); caba.siladji@inmes.rs (C.S.); lazar.milojevic@inmes.rs (L.M.); damjan.gavrilovic@inmes.rs (D.G.); dunja.videnovic@inmes.rs (D.V.); jelena.jovanovic@inmes.rs (J.J.)

**Keywords:** validation, thermal process, *p* value, pasteurization, finely minced cooked chicken sausages, polyamide casing

## Abstract

Current thermal validation in food engineering commonly depends on rigid, temperature-only endpoints that generate thermal stress and the associated energy cost in the case of high-caliber matrices. To overcome this, this study provides a proof of concept for post-heating cooling inertia as the main kinetic driver of cumulative integrated lethality in mass-transfer-limited geometries (*p* value framework), followed by separate enzymatic and not microbial (or, e.g., oxidative) degradation following product heat transfer. Time-series and multi-spatial thermodynamic profiling was performed for the commercial validation runs of three pasteurization processes of large-diameter (90 mm) finely minced cooked chicken emulsions packed in polyamide casing. When active heating was stopped at a lower core limit of 71 °C, the thermal cooling inertia led total integrated lethality to increase by more than 216%, leading to an ultimate core *p* value of 76.87–89.56 min. It systematically exceeded the mandatory food safety limit (*p* ≥ 40 min) in all spatial coordinates in the forced convection chamber, confirming absolute thermal homogeneity. In a subsequent cold-chain stability challenge at around 70 days (0–4 °C), foodborne pathogens were not detected in the product, and saprophytic microflora was limited below critical spoilage bounds (total viable counts (TVC) and dominant lactic acid bacteria (LAB) progressively increased to 3.7 and 3.5 log10 CFU/g on day 70). The autoxidation pathways for primary and secondary lipids remained low (peroxide at 0.00 mmol/kg and malondialdehyde at less than 0.15 mg MDA/kg). On the other hand, a linear progression of free fatty acids (*R^2^* = 0.9673) determined that the single enzymatic lipid hydrolysis is responsible for the final biochemical limiting effect of the sensory lifespan. Shelf life evaluation via rigorous quantitative descriptive analysis showed that all organoleptic attributes remained well above market acceptance criteria until day 70. At the same time, this study supports a scalable, prediction-based thermodynamic paradigm that ensures biological safety and fundamentally optimizes industrial energy use.

## 1. Introduction

Pasteurization is a physical method of food preservation using a temperature lower than the boiling point of water. Pasteurized meat products are classified as highly perishable foods, with physicochemical properties (a_w_, pH) that allow the growth of microorganisms. From a food safety assurance perspective, pasteurization is of particular importance for emulsified (finely minced) cooked chicken sausages packaged in polyamide casings. Within the hazard analysis of this product category, the thermal processing stage, specifically at the pasteurization level, is mandatorily identified as a Critical Control Point (CCP). Hazard Analysis and Critical Control Point (HACCP) is a globally recognized food safety system that prioritizes preventive measures. The food business operator (FBO) must ensure and document this processing stage. Thermal processing remains one of the most efficient techniques for eliminating unwanted microorganisms in food products. The application of heat serves to deactivate pathogens while also contributing to the development of characteristic flavors, aromas, textures, and colors found in cooked dishes [1]. The elimination of microorganisms through heat treatment is influenced by both the temperature and duration of heating. The maximum level of destruction relies on factors such as the inherent resistance of the microorganisms, their population size, reproductive stage, and the characteristics of the substrate involved [2]. The pasteurization value (*p* value) is derived from the lethality measured at the lowest temperature of a product during heat treatment. Pasteurization is confirmed by monitoring F_70_ values, utilizing temperature readings that exceed 55 °C at the geometric center of the meat product [2], and the selected safety threshold (*p* ≥ 40 min) is based accordingly. Inactivation kinetics of *Enterococcus faecium*, a highly heat-resistant reference organism, guarantees the elimination of less tolerant pathogens like *Listeria monocytogenes* and *Salmonella* spp. Achieving this cumulative core value serves as a verified CCP that ensures regulatory compliance and prevents pathogen resurgence during the shelf life period. To establish the pasteurization value limit (*p* value), one must assess how long it takes to achieve a one log cycle reduction in the specified microorganism’s population, assuming an average contamination level of 10^6^ CFU/g, multiplying this figure by the product’s mass and expressing the outcome in minutes [3]. The *p* value is analogous to the F value, but uses a reference temperature of 70 °C with a z value of 10 °C. It is increasingly regarded as a more relevant measure for moderate heat treatments than the traditional F_0_ (121.1 °C, z = 10 °C) used in sterilization validation. The z value signifies the necessary temperature increase for a tenfold (1 log) decrease in a microorganism’s decimal reduction time (D-value) during pasteurization [4]. By integrating *p* values into the optimization process, it can be verified that the intensity of pasteurization is sufficient and that safety is maintained under designated storage conditions. This method also facilitates comparisons of heat-treatment levels across various products. Consequently, FBOs are now able to oversee and regulate each phase of thermal processing effectively, ensuring stringent control throughout and confirming the safety of finely minced cooked sausages within all sections of the pasteurization chamber.

In a safety assurance context, there are regulatory regimes regarding the absence of vegetative pathogens (e.g., *Listeria monocytogenes*, *Salmonella* spp.) from ready-to-eat (RTE) meat commodities [5,6]. In high-caliber vacuum-packaged emulsified matrices, full moisture retention of the formulation results in high water activity (a_w_ > 0.92) and an optimum pH ≥ 5.0 [7]. This peculiar biochemical microenvironment poses a grave threat to food safety, because in response to poor processing, psychrotrophic pathogens may survive and grow after thermal treatment. Hence, systemic biological safety for these mass-transfer-limited geometries remains entirely a function of the exact nature of the thermal pasteurization cycle. To support these thermal interventions without introducing aggressive-pathogenic organisms to industrial plants, processing regulations require modeling inactivation kinetics with a reference surrogate that is very heat-resistant, *Enterococcus faecium* (D_70_ = 2.95 min) [2,3]. By comparing cumulative thermal integrated lethality (*p* value framework) to this target surrogate, we implicitly secure the total elimination of less heat-tolerant vegetative pathogens during the industrial cycle, thereby securing the baseline concern for multi-hurdle preservation architecture.

The shelf life of meat products typically involves detailed and labor-intensive procedures, along with sensory evaluation techniques [8]. During distribution, meat products undergo changes over time—even when stored under cold chain conditions—leading to degradation due to microbial activity and biochemical processes [9]. Consequently, enzymes and bacteria facilitate the breakdown of carbohydrates, proteins, and fats into compounds such as acetaldehyde, hydrogen sulfide, and ammonia, resulting in the generation of various gases throughout the degradation process [10]. The temperature of the meat affects its texture, leading to protein denaturation and cooking loss [11], so it is important to establish metrics to reduce the thermal degradation of sensitive proteins in chicken meat. To determine product shelf life, FBO, in collaboration with relevant professional and scientific institutions, organizes shelf-life studies. The product is subject to targeted and planned testing throughout its shelf life to establish its safety and overall condition by examining the microbiological, physicochemical, and sensory parameters.

To address these fundamental knowledge gaps, this work presents a methodological paradigm in thermal validation that moves from rigid, temperature-only endpoints to dynamic, thermally integrated lethality assessment using the pasteurization (*p*) value in large-diameter food matrices. Instead of establishing a common compliance validation, the present study delineates the major thermodynamic relationship between matrix geometry and post-heating cooling inertia, thereby demonstrating how an integrated kinetic model will both ensure a 70-day shelf-life stability and revolutionize industrial energy efficiency. Based on these combined thermodynamic and biochemical parameters, we establish two alternative scenarios: (1) that in emulsified chicken matrices with high caliber (90 mm) the cooling inertia of the post-heating process is the primary source of thermal lethality (with over 50% of the cumulative *p* value), which allows for active thermal termination of chicken matrices when core temperature is reduced to 71 °C, without compromising the necessary margin of food safety; (2) the ultimate shelf life limit of matrices pasteurized under this refined kinetic tracking mechanism is determined not by microbial resurgence or secondary lipid autoxidation in a 70-day cold-chain storage period, but rather by discrete enzymatic lipid hydrolysis. By systematically showing these phenomena, this study goes beyond site-specific process validation. Instead, it offers a scalable thermodynamic model and rigorous methodological framework that is part of the broader food science literature, radically altering the way CCPs are quantified, modeled and optimized in mass-transfer-limited food geometries.

The unresolved challenges within mass-transfer-limited food geometries and the challenges we face in this study focus on two fundamental scientific questions: First, is the thermodynamic cooling phase mathematically formalizable as a predictable driver of integrated lethality to safely replace rigid temperature endpoints? Second, what biochemical mechanism underpins the quality degradation baseline once microbial resurgence is fully suppressed? To address these questions, we formulate and test two novel research hypotheses:

**Hypothesis 1 (Thermodynamic).**  *In high-caliber (90 mm) emulsified chicken matrices, post-heating cooling inertia dominates the pasteurization stage; over 50% of the total cumulative integrated lethality (p value) is contributed by post-heating cooling inertia, playing an important role in pasteurization efficacy, enabling active thermal termination at a reduced core threshold of 71 °C while systematically exceeding the non-negotiable food safety margin (p ≥ 40 min).*

**Hypothesis 2 (Biochemical).**  *Finely minced matrices pasteurized via this optimized dynamic kinetic tracking will have shelf-life restricted by a mechanism driven by discrete, localized enzymatic lipid hydrolysis, rather than saprophytic microbial proliferation or secondary lipid autoxidation during a 70-day cold-chain storage period, which holds profound strategic and commercial significance for the FBO.*

Verification Logic: This verification matrix to validate these hypotheses is built on a multi-hurdle experimental architecture. To prove the validity of their claims, Hypothesis 1 is verified through real-time, multi-spatial thermodynamic profiling (three independent validation runs across vertical chamber tiers) and one-way ANOVA to ensure spatial thermodynamic homogeneity. Hypothesis 2 is confirmed with precision experiments tracking synchronized kinetic parameters during a 70-day stability challenge (0–4 °C) under cold-chain conditions, based on high-precision quantitative descriptive sensory analysis, TVC, psychrotrophic microflora kinetics, and continuous linear regression modeling of free fatty acid accumulation versus primary and secondary autoxidation markers.

## 2. Materials and Methods

To clarify the overall workflow and structure of the study, we created a phase diagram of the development process, illustrating the steps taken and the methods applied.

**Figure 1:**
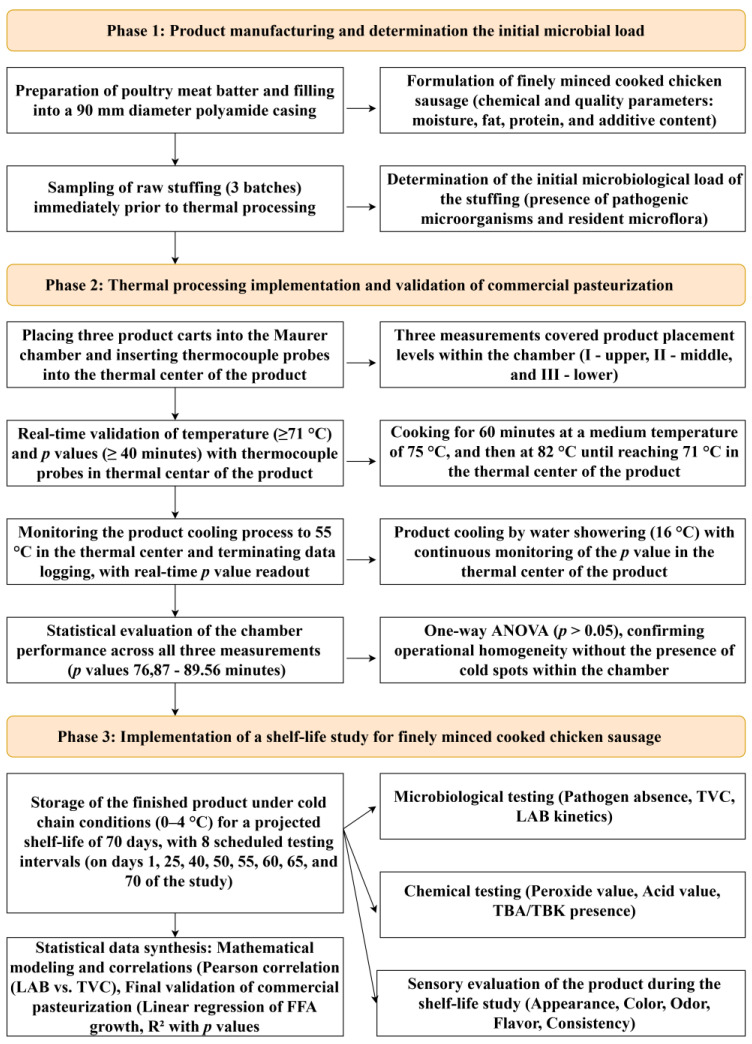
Schematic flowchart of the experimental design and methodological workflow, illustrating emulsion preparation, thermal validation kinetics inside the Maurer chamber, multi-hurdle preservation monitoring during the 70-day cold storage, and statistical analysis pathways.

The commercial pasteurization and the corresponding shelf life of wide-diameter emulsified finely minced cooked chicken sausages were extensively validated using a systematic, four-phase experimental matrix. An investigation integrated raw matrix characterization, real-time thermodynamic profiling of thermal kinetics, cold-chain stability challenge tracking, and advanced statistical modeling. This multi-hurdle method was devised to identify and quantify the key underlying drivers of microbial inactivation and biochemical degradation during a 70-day storage timeframe. The comprehensive experimental architecture and procedural workflow of this study are visually mapped out in Figure 1.

### 2.1. Phase 1: Product Manufacturing and Determination of Initial Microbial Load

The making of finely minced cooked chicken sausages at the FBO production facility is a specific technological process, and all technologies are aligned with current legal requirements and quality standards. The manufacturer’s specification, a legal, technical document that defines the organoleptic, chemical, and rheological properties of the final product, provides a basic framework of the industrial manufacturing of this product category. The standardized formulation of the finely minced cooked chicken sausage consisted of a meat matrix comprising mechanically deboned chicken meat (45.0%, *w*/*w*), first-category chicken meat (25.0%, *w*/*w*), and chicken skins (5.0%, *w*/*w*), with the addition of ice water (20.0%, *w*/*w*). To optimize the rheological properties and water-binding capacity, non-meat functional ingredients were incorporated, including rice flour (2.0%, *w*/*w*), potato starch (0.5%, *w*/*w*), and pea protein isolate (0.5%, *w*/*w*). The additive profile and spice blend (1.0%, *w*/*w*) included dextrose (0.5%, *w*/*w*), stabilizers (diphosphates and triphosphates, E450 and E451), thickeners (sodium alginate and carboxymethyl cellulose, E401 and E466), acidity regulators (sodium acetates and calcium sulfate, E262 and E516), an antioxidant (ascorbic acid, E300), and a preservative (sodium nitrite, E250). From the analytical specification provided by the produce company, the nutritional content of the product shows an energy value of 786 kJ (182 kcal) per 100 g, a lipid fraction of 16.7 g, and saturated fatty acids of 4.4 g in weight. The protein content is 11 g, and the carbohydrate fraction is 2.0 g, of which simple sugars are 0.8 g. The sodium chloride content, expressed as salt, is determined to be 1.7 g. Moisture content is 66%.

The first and necessary part in the raw material value chain is raw materials selected and provided with careful scrutiny on freshness and microbiological status, with pH values under strict monitoring, including mechanically deboned chicken meat. The regulation of temperature of raw materials during storage and preservation of those materials under cooling or freezing conditions directly contributes to the stability of the next emulsion system. The technological process of meat batter manufacturing was executed through a controlled comminution and emulsification sequence in a high-speed vacuum cutter (VEMAG Maschinenbau GmbH, Verden, Germany). Initially, chilled chicken meat (0–2 °C) and partially defrosted mechanically deboned chicken meat (−2 to 0 °C) were placed in the cutter bowl along with nitrite curing salt and phosphates, and comminuted at low speed with the addition of one-third of the water to extract myofibrillar proteins (actin and myosin) while maintaining the mixture temperature below 4 °C. Subsequently, the chicken skin, pea protein isolate, and rice flour were introduced, and the cutter was operated at maximum speed (3000 rpm) to finely reduce fat particles and encapsulate them within the extracted protein matrix, thereby establishing a stable, raw emulsion. In the final stage of cutting, dextrose and the spice blend were added alongside the remaining water, and the process was completed under vacuum to eliminate air pockets until the meat batter reached a temperature of 10–12 °C to prevent emulsion breakdown. The resulting homogeneous, highly viscous composite gel matrix was immediately transferred to a vacuum stuffer, filled under pressure into 90 mm diameter polyamide casings, and securely clipped to produce the sausage links ready for subsequent thermal processing. The pH value of the raw meat batter was 6.21. It was measured directly using a digital penetration pH meter (Model 206-pH2, Testo SE & Co. KGaA, Titisee-Neustadt, Germany) calibrated with standard buffer solutions (pH 4.01 and 7.00) at room temperature. Mechanically deboned chicken meat naturally has a higher pH value, generally between 6.3–6.5, since it consists of bone marrow components and is processed under high pressure compared to intact breast meat. Additionally, alkaline phosphates are used to increase the pH applied in the comminution process to maximise the water binding of the myofibrillar proteins. Also considering that pea protein isolates possess a slightly alkaline to neutral pH (roughly 6.5–7.0), the whole structural matrix is maintained at a stable, elevated pH. This biochemical profile is important to maintain the stability of the meat emulsion before the thermal processing stage. The apparent viscosity of the raw emulsion was assessed at 10 °C using a rotational viscometer (Model DV2T, Brookfield Engineering Laboratories, Middleboro, MA, USA) equipped with a Helipath stand and a T-E spindle spinning at 5 rpm to produce 241 Pa·s, which is because of the non-meat part of the product formulation (rice flour and pea protein isolates). These constituents act as thickeners and hydrocolloids that efficiently bind free water and additional ice content. Moreover, the low temperature at the end of comminution (10–12 °C) ensures a solid formation of lipids from the chicken skin, which increases structural density; myofibrillar proteins build a dense pre-gel matrix.

Prior to the initiation of the commercial pasteurization validation, in order to comprehensively assess the baseline microbial burden of the meat emulsion, planned sampling of the raw batter stuffed into polyamide casings was performed immediately prior to thermal processing across three consecutive production runs. Evaluating the microbiological profile of raw batter prior to pasteurization is not governed by specific regulatory frameworks or prescribed legal thresholds that would justify the a priori rejection of a production batch. Because the product is not intended for consumption in its raw state, this analysis aimed to quantify the initial contamination level and evaluate the microbial risk targeted for inactivation during pasteurization. The samples were subjected to qualitative and quantitative screening for foodborne pathogens, including sulfite-reducing clostridia (*Clostridium* spp.) [12], *Bacillus cereus* [13], *Listeria monocytogenes* [14], *Salmonella* spp. [15], and *Escherichia coli* [16]. Additionally, resident microflora, specifically *Enterobacteriaceae* [17] and TVC [18], were enumerated. All analyses were performed immediately post-sampling in the FBO laboratory utilizing validated, accredited methodologies. The finely minced cooked chicken sausage batter was stuffed into polyamide casings (90 mm diameter) using a vacuum filler. The batter was held for no longer than one hour prior to thermal processing, which is consistent with United States Department of Agriculture (USDA) recommendations [19].

### 2.2. Phase 2: Thermal Processing Implementation and Validation of Commercial Pasteurization

Commercial pasteurization is a production step defined as a CCP in the FBO food safety risk analysis documents in accordance with legal regulations. The validation process was repeated three times in the production plant on three different working days. Three validations were conducted during regular production at the facility, using a Maurer chamber (manufactured by Maurer-Atmos Middleby GmbH, Reichenau, Germany). The chamber has a single door and can accommodate three carts in a row, and it was run through a high-performance vertical cross-flow ventilation system and maintained an average air velocity of 2.5 m/s across the product zones to determine systemic airflow dynamics and loading rigor. The thermal processing unit was configured with a total heating capacity of 45 kW and sustained by a stable saturated steam supply at a regulated pressure of 3.0 bar. All three measurements were conducted under maximum chamber capacity conditions (660 kg), with each of the three carts loaded with 220 kg of product. The sausages were distributed across three distinct vertical tiers (upper, middle, and lower) and suspended from triangular metal sticks in batches of five per row. Each tier accommodated seven sticks, each holding five products, which were strategically spaced to prevent any mutual contact. The mass of each individual product unit was equalized and maintained at 2.0 kg.

Each validation process was performed under identical chamber medium conditions (two-step cooking process: 60 min at a chamber temperature of 75 °C, followed by processing at 82 °C until the product center reached 71 °C). Upon completion of the active heating phase (71 °C in thermal center of the product), the products on carts were immediately subjected to chilling in a storage chamber, and the validation process continued until the temperature dropped below 55 °C in thermal center of the product [2,3,20,21]. During this period, the products were transferred from the chamber to a water spray cooling room. The water spray was properly controlled at an optimal temperature of 16 °C, which is defined according to empirical operations on the basis of the FBO data. This temperature was chosen to keep the cooling rate within a systematic and safe range as this would prevent structural deformation or thermal shock that could be a detriment to the structural integrity and aesthetic uniformity of the ready-to-eat (RTE) product.

The lethality of the thermal process is determined by the formula [2]:

F_s_ = D_r_ (log N_o_ − Log N)

where D_r_ is the decimal reduction time (min) at the reference heating temperature, N_o_ is the initial microbial count, and N is the number of surviving microorganisms (D-concept).

To achieve a scientifically rigorous target safety threshold, the calculation is based on the total microbial load per product unit rather than concentration alone, ensuring compliance with industrial food safety protocols:

Reference Organism: *Enterococcus faecium*

Initial Concentration (N_0_: 10^5^ CFU/g).

Total Unit Mass: 2000 g (2 kg sausage unit).

Total Initial Load per Unit (N_0,total_): 10^5^ CFU/g × 2000 g = 2 × 10^8^ CFU/unit.

Target Performance Standard: To ensure safety across large-caliber matrices, a stringent 12D reduction concept (analogous to the botulinum cook standard but adapted for highly resistant vegetative reference surrogates in pasteurization) or a strict target survival probability of N = 10^−5^ CFU/unit was applied to clear the initial (2 × 10^8^) total load.

Using the standard log-linear thermal death kinetic formula:

P_target_ = D_70_ × (log N_0,total_ − log N_final_)

P_target_ = 2.95 × (log (2 × 10^8^) − log (10^−5^))

P_target_ = 2.95 × (8.301 − (−5)) = 2.95 × 13.301 = 39.238 min.

This mathematically justifies the rounded operational safety threshold of (*p* ≥ 40 min) used for routine CCP monitoring.

As the primary spoilage microflora in pasteurized meat products, *Enterococcus faecium* exhibits superior heat resistance compared to vegetative pathogenic bacteria, with a reported decimal reduction time of D_70_ = 2.95 min. Choosing the critical processing limit (*p* ≥ 40 min) is directly informed by the non-linearity of thermal death kinetics of *Enterococcus faecium*, which serves as the internationally recognized reference surrogate for vegetative pathogen validation [2,22,23]. This rounded limit introduces an additional safety buffer that safely accommodates any potential minor localized heat transfer delays within the high-caliber matrix. Assuming an initial baseline contamination of *Enterococci* of N_0_ = 10^5^ CFU/g, within a 2 kg (2000 g) individual sausage link, the total initial bioburden equals 2 × 10^8^ CFU per product unit. To achieve an ultra-conservative safety margin under industrial conditions, a total target reduction of (13.3 log) cycles was applied. Based on the thermal death kinetics of *Enterococcus faecium* (D_70_ = 2.95 min), the required thermal process lethality (F_70_) is mathematically determined to be 39.238 min. Consequently, for operational standardization and routine CCP monitoring, the target *p* value safety threshold was established at a rounded value of 40 min.

The technological production steps are synchronized, ensuring that thermal processing does not represent a production “bottleneck”, which is often the case with FBOs in Serbia. Validation was performed using the E-Val Pro thermocouple (manufacturer Ellab A/S, Hillerød, Denmark), serial number 411982, with validated software—US FDA, 21 CFR part 11, GMP, ver. 4.6.1.0. Ellab ValSuite Pro software, ver. 5.2.015, was used to generate the technical report for each measurement.

In all three measurements, four thermocouple probes with compensation cables were used. To ensure measurement accuracy, the thermocouple probes undergo routine calibration every three months at the Institute of Physical Chemistry in Belgrade, Republic of Serbia, which issues a formal certificate of successful calibration for each probe utilized in the study. The probes were placed in the thermal center of the product at three points—in the middle of each product cart—while one probe was placed in the chamber medium, in accordance with the Guidelines for Conducting Thermal Validation Studies—Implementation of Penetration Studies [24]. While three probes were placed in thermal center of the product, in each of three measurements, a fourth probe was assigned to monitor the ambient chamber medium, relocated to a different cart during each cycle according to the experimental design. All probe locations were restricted to the central core of the carts to avoid exposure to direct heat waves beneath the exhaust nozzles aligned on both sides of the chamber.

**Figure 2:**
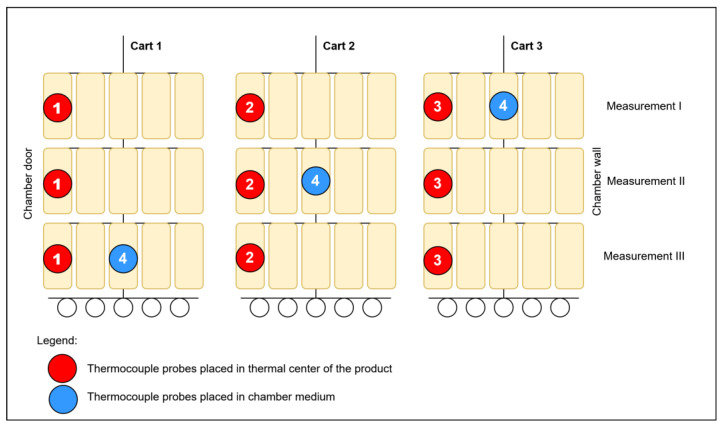
Position of the probes in thermal center of the product (Thermocouple probes 1–3) and in chamber medium (Thermocouple probe 4), on carts during pasteurization in all three measurements seen from the side.

During routine production, commercial pasteurization validation was conducted in three different spatial configurations within the product carts. Measurement I focused on testing the products that were located in the upper tier of the cart. Then Measurement II was to observe the thermal process within the central section, and Measurement III was to ensure the validation of products positioned on the lower tier. This provided the thermal validation of all three loading levels under standard operational conditions. The position of the probes on carts for all three measurements is presented in Figure 1 and Figure 2. To prevent displacement, thermocouple probes were inserted into the thermal center of the product only after the product carts had been loaded into the thermal processing chamber (Maurer-Atmos, Reichenau, Germany). Due to the pasty and low-viscosity consistency of the batter, minor movements could easily cause the probe tip to deviate from the thermal center. To mitigate this, 20-cm long needle probes were utilized, ensuring sufficient depth penetration and secure fixation within the thermal center of the large-diameter (Ø 90 mm) chicken sausage in a polyamide casing. Following insertion, each probe was secured along the vertical axis using plastic cable ties. Meticulous care was taken during this positioning procedure to guarantee the precise alignment of the measuring tip within the central longitudinal axis of the product.

**Figure 3:**
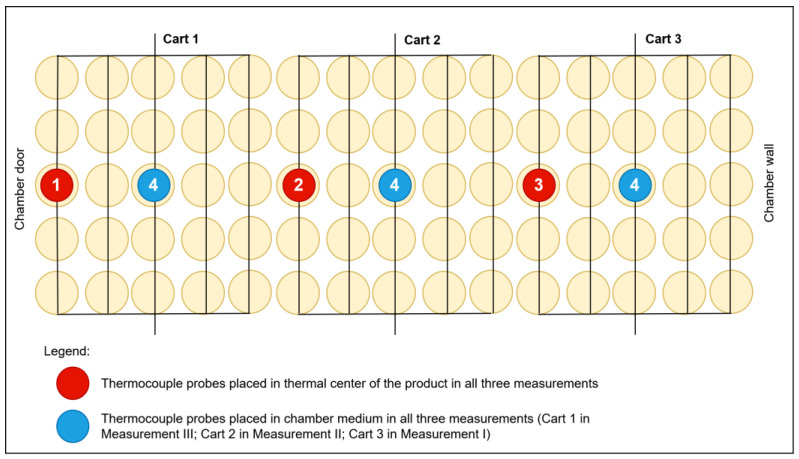
Position of the probes in thermal center of the product (Thermocouple probes 1–3) and in chamber medium (Thermocouple probe 4), on carts during pasteurization in all three measurements seen from above.

During each measurement, temperature and *p* value were monitored in real-time. The *p* value was determined using original Ellab software, which instantaneously calculated the thermal processing lethality unit in real-time and displayed it alongside the thermal center (3 probes) and chamber medium temperature (1 probe). The probes were used as Temperature Measuring Devices (TMD), in accordance with the Guidelines [24] for penetration studies. Temperature and *p* value recordings were logged by the software every minute with a precision of 0.1 °C, a recording method similar to those used in other studies [25]. The spatial distribution of the probes embedded within the products and the carts medium inside the Maurer chamber is depicted in Figure 2 and Figure 3.

For the assessment of operational performance and thermal homogeneity of the processing chamber, we made continuous tests using temperature information generated from the three independent validation runs (Measurements I, II, and III) and performed a one-way analysis of variance (ANOVA). In statistics, the critical thermal parameters for the upper, central, and lower loading tiers did not significantly differ (*p* > 0.05). This equivalence unambiguously verifies the uniformity of operation of the system under standard production conditions.

To ensure a robust experimental design, probe allocation was directly predicated on the thermodynamic and aerodynamic profile of the Maurer pasteurization chamber. This specific equipment utilizes a series of lateral-upper evaporator/heating ducts that actively discharge high-temperature saturated steam. The forced convection matrix generates a distinctive cyclonic airflow pattern: the thermal medium is driven downward along the chamber boundary walls, deflects off the floor plane, and subsequently ascends vertically through the geometric center of the product carts before completing the circuit at the ceiling canopy. Consequently, product units positioned at the periphery of the carts experience direct thermal impingement and accelerated heat transfer rates. Conversely, the core section of the product mass is shielded from this primary convective flow, establishing it as the mass-transfer-limited zone (‘worst-case scenario’). To rigorously guarantee the microbiological safety of the entire production batch, high-precision core temperature sensors were deliberately embedded within the thermal center of sausages located exclusively inside this low-energy central matrix. Validating thermal adequacy under these restrictive boundaries ensures that acceptable lethality values (P_70_) inherently translate to absolute safety across all non-critical zones of the chamber. Moreover, the consistent and uniform temperature distribution observed throughout the thermal cycle confirms the spatial thermodynamic homogeneity of the chamber environment. This uniform thermal delivery ensures a highly stable, reproducible, and reliable commercial pasteurization process across the entire product batch.

### 2.3. Phase 3: Implementation of Shelf-Life Study for Finely Minced Cooked Chicken Sausage

Validating pasteurization through a shelf-life study is crucial because it confirms that the process is not only theoretically sound but also practically effective over time. Establishing the limit of acceptability is indeed the most difficult step, as it requires balancing food safety and product quality. To achieve this, planned testing (microbiological, chemical, and sensory) was organized throughout a projected shelf life of 70 days. This estimated period represented the FBO target, intended to facilitate their own production and turnover planning. A total of 8 planned tests were conducted (on days 1, 25, 40, 50, 55, 60, 65, and 70 of the shelf life). Each planned test consisted of three segments (microbiological, chemical, and sensory). Following production, the product was stored on the FBO premises and was sampled and analyzed in the laboratory according to the previously established schedule.

The pH value of the pasteurized chicken sausage core matrix was determined via direct potentiometric insertion using a high-precision digital penetration pH meter (Model 206-pH2, Testo SE & Co. KGaA, Titisee-Neustadt, Germany) equipped with a robust, plastic-sheathed penetration probe and an integrated temperature sensor. Prior to each batch analysis, the instrument underwent a three-point calibration protocol at room temperature using certified standard technical buffer solutions (pH 4.01, 7.00, and 10.01; Testo SE & Co. KGaA). The water activity (*a*_w_) of the finely minced emulsion was quantified using a sophisticated chilled-mirror dew point water activity meter (Model AquaLab 4TE, Meter Group, Inc., Pullman, WA, USA) with a resolution of ±0.003 *a*_w_. Calibration was systematically verified using primary salt slurry standards (0.250, 0.500, 0.760, and 0.984 *a*_w_ NaCl/LiCl solutions provided by the manufacturer) at a strictly controlled chamber temperature of (25.0 ± 0.1 °C). All physicochemical measurements were executed in five independent replicates (n = 5) per sampling interval.

In accordance with the predefined sampling timeline, microbiological analyses were performed at each designated shelf-life interval. The screening encompassed foodborne pathogens, including sulfite-reducing clostridia (*Clostridium* spp.) [12], *Listeria monocytogenes* [14], *Salmonella* spp. [15], coagulase-positive staphylococci [26], *Escherichia coli* [16], and *Campylobacter* spp. [27]. Additionally, microbial spoilage indicators, specifically *Enterobacteriaceae* [17], LAB [28], and TVC [18], were quantified. The plotted data points represent the mean values of five sample units evaluated at each designated testing interval. Pathogen-specific thermal death kinetics, derived from the exact product matrix under evaluation, must be utilized to accurately determine thermal process lethality for fully cooked, vacuum-packaged chicken products [29].

In accordance with the predefined sampling timeline, lipid hydrolytic and oxidative stability parameters were monitored at each designated shelf-life interval. Lipid degradation was evaluated through the determination of acid value [30], peroxide value [31], and malondialdehyde (MDA) content, the latter quantified via the 2-thiobarbituric acid (TBA) assay [32,33]. 

To assess consumer acceptability of the chicken sausage produced via a validated commercial pasteurization process, a comparative sensory evaluation was performed. Sensory profiling was conducted within an ISO/IEC 17025 [34] accredited laboratory environment by a panel of five validated experts (including two institutional specialists who co-authored the broader processing study). To eliminate cognitive bias and guarantee the strict independence of the sensory data, a robust triple-blind experimental design was enforced. Samples were completely anonymized using randomized three-digit alphanumeric codes, prepared by an external technician, and evaluated in isolated sensory booths under standardized lighting conditions. Panelists were completely blinded to the thermal processing variables (*p* values) and storage intervals. The utilization of a specialized, highly calibrated five-member expert matrix aligns with established standards—ISO 6658:2017 [35]. Small, accredited panels provide highly reproducible quality limits that minimize the subjective variance inherent in larger consumer cohorts.

Product characterization was conducted by a selected panel of five trained experts, whose sensory acuity had been verified through standardized screening protocols [36,37,38,39]. The sensory attributes of the product were evaluated via Quantitative Descriptive Analysis (QDA) in accordance with the ISO 6658:2017 standard [35]. The sensory evaluation framework was designed using the ISO 6658:2017 standard with a 5-point numerical-descriptive scale (Table 1). A score equal to 2.0 was chosen as the cut-off point for market acceptability and presented in Table 1. Data are expressed as the mean scores derived from the five independent panelists.

Various statistical methods are utilized to confirm the commercial pasteurization process and evaluate the shelf life of the product in order to guarantee food quality and safety. A one-way ANOVA is employed to reveal significant differences between the mean contamination levels of different batches of raw meat batter and to check homogeneity of heat distribution of the pasteurization chamber using *p* values at different vertical locations. The strength as well as direction of the linear relationship between growth of LAB and TVC are calculated using Pearson’s Correlation Coefficient. Descriptive statistics of the microbial counts, chemical parameters, and sensory analysis scores are given, including the mean, standard deviation, and range values. A statistical test for significance is conducted based on *p*-values. Kinetic analysis is performed by plotting data over time, while linear regression (*R*^2^ value) is used for the growth trend estimation of free fatty acids.

## 3. Results

### 3.1. Microbiological Profile of the Meat Batter Prior to Thermal Processing

The results of the targeted microbiological profiling of the meat emulsion prior to thermal processing are presented in Table 2.

One-way ANOVA revealed no statistically significant differences (*p* > 0.05) among the mean contamination levels of the evaluated batches, confirming a high degree of homogeneity and structural consistency within the raw batter prior to pasteurization.

### 3.2. Validation of Commercial Pasteurization

**Figure 4:**
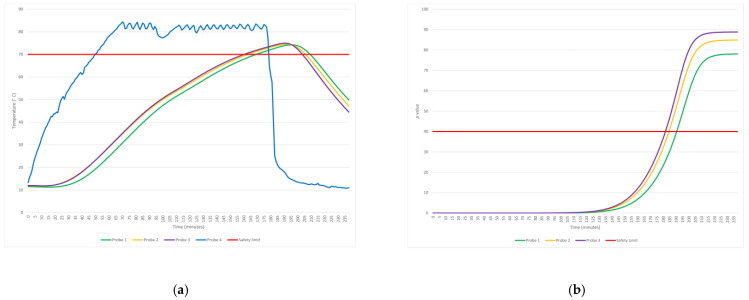
First measurement of *p* values and temperature in thermal center of the product and in the chamber medium: (**a**) temperature in thermal center of the product and in the chamber medium; (**b**) *p* value in thermal center of the product.

**Figure 5:**
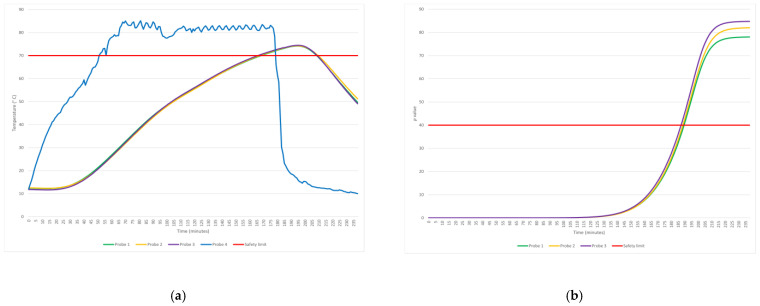
Second measurement of *p* values and temperature in thermal center of the product and in the chamber medium: (**a**) temperature in thermal center of the product and in the chamber medium; (**b**) *p* value in thermal center of the product.

**Figure 6:**
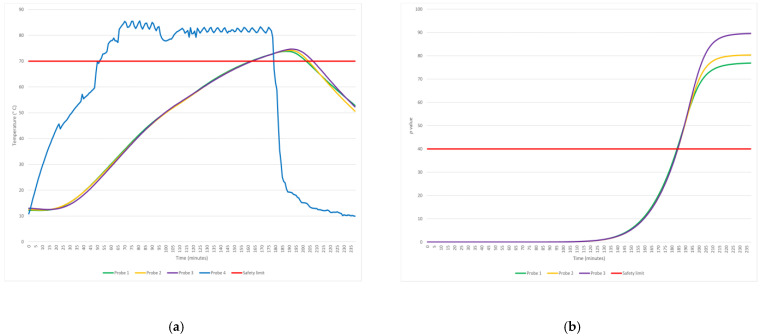
Third measurement of *p* values and temperature in thermal center of the product and in the chamber medium: (**a**) Temperature in thermal center of the product and in the chamber medium; (**b**) *p* value in thermal center of the product.

The validation protocol was monitored in real time, with temperature and pasteurization values (*p* values) continuously recorded from the equipment display during all three independent measurement cycles. All recorded measurement data are provided in Supplementary Tables S1–S3. The corresponding thermal profiles and inactivation dynamics are illustrated in Figure 4, Figure 5 and Figure 6.

The validation of the chicken sausage pasteurization process was performed during routine manufacturing under operational parameters defined by the thermal treatment program. The datasets from all three measurement cycles are illustrated in Figure 3, Figure 4 and Figure 5. In each figure, the primary y-axis (left) displays the product thermal center temperature during pasteurization, plotted against the processing time in minutes (x-axis), whereas the secondary y-axis (right) depicts the cumulative pasteurization value (*p* value) accumulation in the product thermal center throughout the thermal process.

Product microbiological safety was consistently verified across all three independent measurement cycles, with core *p* values strictly exceeding the critical threshold of 40 min. The intermediate *p* values recorded at the onset of the chilling phase (the 178th minute of the thermal cycle, coinciding with a core temperature of 70 °C), along with the definitive cumulative *p* values obtained at the conclusion of the validation protocol (the 238th minute), are summarized in Table 3.

Statistical analysis revealed that the variations in *p* values at the end of the active heating phase among the upper, middle, and lower probe positions within the chamber (*p* = 0.20) were not significant (*p* > 0.05). This homogeneity confirms a uniform heat distribution throughout the chamber volume during the active thermal phase, irrespective of the vertical placement of the product. Similarly, the differences in cumulative *p* values recorded at the conclusion of the measurement cycle among the upper, middle, and lower vertical positions (*p* = 0.54) demonstrated no significant variance (*p* > 0.05). The comparative analysis regarding the differences in the achieved mean *p* values at the end of the active heating phase and at the final conclusion of the measurements cycle is summarized in Table 4.

Following the cessation of the active heating phase, *p* value increased markedly by +56.77 ± 3.63 min, representing a relative expansion of 216.2 ± 34.87%. Due to the slow cooling rate of the product interior, the thermal center remained within the lethal pasteurization temperature range for an extended duration, persisting even after the chamber operations had ceased.

### 3.3. Shelf Life Testing

#### 3.3.1. Microbiological Analysis

**Figure 7:**
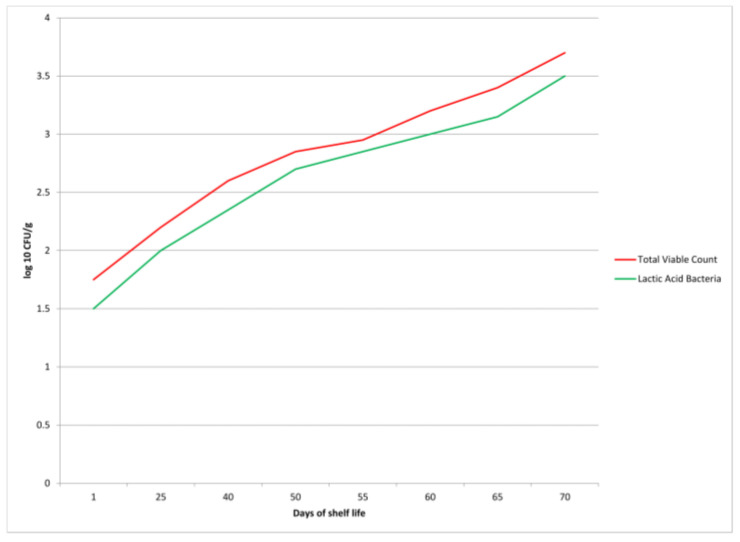
Growth and survival of the determined microorganisms in the product during the shelf-life study.

The growth and survival data of the determined microorganisms during the shelf-life study are presented in Figure 7.

Microbiological analysis indicated that *Salmonella* spp. and *L. monocytogenes* were not detected from samples throughout the testing period. Furthermore, counts of *Campylobacter* spp., coagulase-positive staphylococci, *E. coli*, *Enterobacteriaceae*, and sulfite-reducing clostridia remained below 10 CFU/g. The total plate count (TVC) showed a gradual increase from day 1 to day 70, reaching a maximum of 3.7 log10 CFU/g on day 70 of the stability study. Similarly, LAB counts increased progressively over the 70-day period, peaking at 3.5 log10 CFU/g on the final day. Pearson’s correlation coefficient (r = 0.993) demonstrated a strong, statistically significant positive correlation (*p* < 0.001) between LAB growth and the TVC.

#### 3.3.2. Chemical Analysis

**Figure 8:**
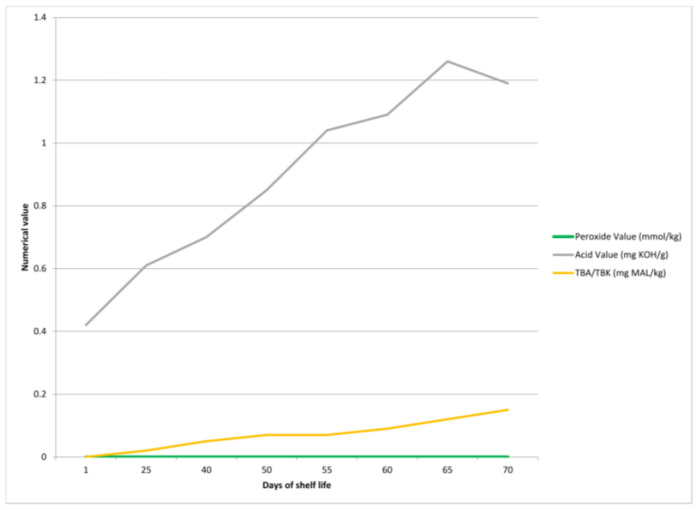
Changes in peroxide value, acid value, and malondialdehyde content in the product during the shelf-life study.

The variations in peroxide and acid values, as well as the presence of malondialdehyde in the product during the shelf-life study, are presented in Figure 8.

The peroxide value remained at 0.00 mmol/kg from day 1 to day 70 of the shelf-life study. The acid value, as a measure of lipid hydrolysis, ranged from 0.42 mg KOH/g (day 1) to 1.19 mg KOH/g (day 70). The malondialdehyde content, determined as thiobarbituric acid reactive substances (TBARS), ranged from 0.00 mg MDA/kg on day 1 to 0.15 mg MDA/kg on day 70.

#### 3.3.3. Sensory Analysis

**Figure 9:**
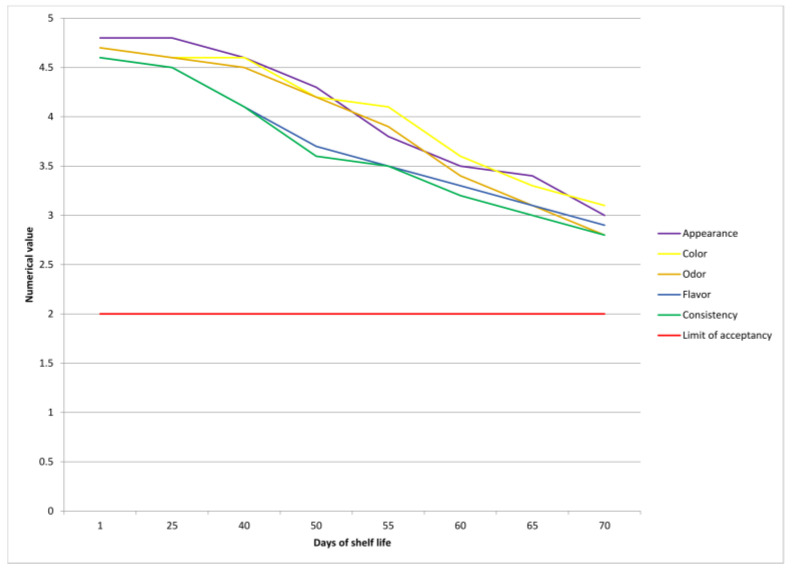
Sensory evaluation of the product during the shelf-life study.

Sensory attributes (appearance, color, taste, odor, and consistency) of the finely minced cooked chicken sausage product were evaluated during the 70-day shelf-life study. The mean scores are presented in Figure 9.

From day 1 to day 55 of the shelf-life study, the appearance, odor, color, taste, and consistency of the finely minced cooked chicken sausage were evaluated as ‘exceptionally acceptable’ and ‘highly acceptable’. From day 60 to day 70 of the study, all evaluated sensory attributes were scored as ‘acceptable’.

#### 3.3.4. Water Activity (a_w_) and pH Testing of Wide-Diameter Finely Minced Cooked Chicken Sausages Packed in Polyamide Casing

The longitudinal variations in water activity (*a*_w_) and pH values of wide-diameter finely minced cooked chicken sausages packed in polyamide casing, throughout the 70-day cold-chain storage period (0–4 °C) are compiled in Table 5.

## 4. Discussion

Thermal processing represents a fundamental method in food preservation; the application of thermal energy inactivates microorganisms and enzymes, thereby directly enhancing food safety and shelf life [40]. Modern lifestyles dictate an increased demand for ready-to-eat (RTE) meat products. Consequently, commercially available meat products are expected to be minimally processed while remaining stable under defined storage and distribution conditions. While pasteurization extends shelf life by eliminating vegetative pathogenic microorganisms and reducing the overall microbial load of the sausage batter relative to raw materials [41], suboptimal thermal treatment can compromise protein structure and degrade vital nutrients [42]; consequently, this process must be rigorously synchronized with documented food safety management systems to maximize production control and guarantee final product safety while optimizing energy consumption [43]. Particular attention is focused on the elimination of pathogenic microorganisms, such as *Salmonella* spp., *Listeria monocytogenes*, and *Escherichia coli* [44,45,46]. Spoilage bacteria, such as *Enterococcus* spp., *Lactobacillus* spp., and *Micrococcus* spp., exhibit higher thermal resistance than pathogens like *Salmonella* spp. and *Staphylococcus* spp.; consequently, due to its superior heat tolerance relative to pathogenic microorganisms, *Enterococcus faecium* is widely utilized as a reference organism for evaluating pasteurization efficiency [2,23,24], whereby the *p* value concept is based on the inactivation kinetics of this specific, highly heat-resistant microorganism. Pasteurization is designed to eliminate vegetative microorganisms and spoilage agents rather than to achieve complete microbial inactivation [47]. Commercial stability is a multi-hurdle preservation system: although the cumulative *p* value eliminates vegetative pathogens, the continuous cold-chain maintenance at 0–4 °C serves as a critical subsequent hurdle that suppresses any surviving microflora over the 70-day timeframe. Any process deviation is considered non-compliant, necessitating the withdrawal and appropriate disposal of affected batches [48], while effective implementation of HACCP principles and utilization of microbiological data acquired during process validation is paramount, serving as a framework to optimize existing control measures [49].

The meat industry continuously develops strategies to ensure product safety, focusing on models that primarily guarantee the shelf-life of pasteurized products under defined cold chain storage conditions, while optimizing sensory attributes and minimizing energy consumption. However, establishing higher validation temperatures, such as 74 °C at the thermal center—especially during the processing of large-diameter meat products—raises concerns regarding overall process control. Near the end of the thermal cycle, the core temperature exhibits slow growth dynamics. This results in unnecessary exposure to elevated temperatures, degradation of nutritional properties, and a higher carbon footprint, while the lethal effect of the temperature during the post-heating phase is typically neglected. This approach could be advanced by validating commercial pasteurization using *p* values, which incorporate the lethal effect of the cooling phase, particularly for large-diameter products. Terminating the heating phase at 71 °C, once the legally prescribed limit is achieved, would significantly shorten the process, which would otherwise be unnecessarily prolonged to reach 74 °C. Relevant EU Regulation [50], rather than prescribing a single fixed parameter, outlines a scale of equivalent time-temperature combinations, whereby increasing processing temperatures linearly reduces the required holding time within the thermal core of the product. In larger-diameter products, the residence time of the product mass at elevated temperatures is inherently prolonged; consequently, utilizing processing temperatures lower than the maximum prescribed 74 °C within the thermal core is highly advisable. Analysis of the results indicates that the maximum *p* values at the end of the process (Table 3) approach 76.87–89.56 min, which is twice the required safety limit (≥40 min). This demonstrates that the product received more than sufficient thermal energy at every point within the chamber to eliminate target microorganisms. Furthermore, ANOVA testing for both phases revealed no statistically significant differences (*p* > 0.05), confirming spatial thermodynamic homogeneity inside the chamber that could compromise any part of the production batch during pasteurization. The validation process of this commercial pasteurization is stable and highly reproducible. During the chilling phase of finely minced cooked chicken sausages packaged in polyamide casings, the application of moderate-temperature cooling water (potable water up to 16 °C) is recommended [51]. Utilizing cooling water at lower temperatures can induce longitudinal casing deformation and wrinkling, potentially resulting in commercial product withdrawal. Concurrently, these findings highlight opportunities for technological optimization, specifically by shortening the temperature-holding phase to preserve sensory quality. These approaches define the direction for future research and industrial implementation.

At the cessation of the active heating phase (178 min), the target safety threshold of *p* ≥ 40 min was not achieved at any monitored position (Table 3). For large-diameter products (diameter 90 mm), the cumulative pasteurization efficacy heavily relies on cooling inertia, which secures the mandatory safety margins and drives *p* values substantially beyond the required limit. The significant absolute rise in the pasteurization value ΔP = 56.77 ± 3.63 min upon heating reinforces a crucial thermodynamic process that goes beyond common factory compliance, representing a 216.2 ± 34.87% increase (Table 4). Typically, processing in industrial validation is terminated as soon as a target endpoint (74 °C) is reached, thus using real-time core temperature monitoring with traditional methods. This simplistic, dichotomous methodology completely overlooks the substantial thermal energy derived from the cooling path. In mass transfer-limited food geometries such as the 90 mm diameter matrices considered here, the slow heat dissipation becomes a dominant kinetic driver, accounting for more than 50% of the total cumulative integrated lethality. By changing the validation paradigm from a temperature-only-standard end-point to dynamic and integrated thermal kinetics *p* value tracking, this work gives us a portable and scalable framework for complex meat emulsions. Thus, quantifying this thermodynamic inertia allows food systems operators to systematically reduce peak thermal stress, thus reducing active heating cycles without compromising non-negotiable microbiological safety margins. The validation of active heating phase at a core temperature of 71 °C and yielding final *p* values of 76.87–89.56 min compares favorably with traditional validation data for small-diameter frankfurter-type sausages; the latter required heating to 74 °C to achieve *p* values of 61.45–81.07 min [43]. In small-diameter matrices, the rapid core temperature decline during cooling abruptly halts further *p* value accumulation, a thermodynamic behavior distinctly absent in large-diameter products.

Analysis of the microbiological profile of the sausage batter immediately prior to thermal processing revealed that the total aerobic colony count (TVC) fell within a narrow range, from 6.95 ± 0.81 log CFU/g (Measurement II) to 7.15 ± 0.74 log CFU/g (Measurement III). This high initial contamination level (>7 log CFU/g) is typical for finely minced, mechanically separated, and technologically manipulated chicken meat prior to heat treatment [52]. The mean counts of *Enterobacteriaceae* ranged from 2.82 ± 0.54 to 3.01 ± 0.36 log CFU/g. The presence of this bacterial family at a level of approximately 3 log CFU/g serves as a key hygiene indicator, reflecting contamination risks during slaughtering, evisceration, and primary carcass deboning. Concurrently, the mean contamination level of *Escherichia coli* in the batter varied between 2.37 ± 0.85 and 2.53 ± 0.95 log CFU/g, indicating a stable, moderate presence of fecal indicators originating from the raw materials, which is common in industrial meat processing before lethal temperature exposure. A comparative analysis revealed no statistically significant differences (*p* > 0.05), which indicates that the mixing, emulsifying, and batter preparation processes in the bowl cutter were highly uniform and consequently, the microbial load was homogeneously distributed throughout the entire matrix. Due to the high initial load of saprophytic microflora within the meat batter, restricting the pre-pasteurization holding time to a maximum of two hours is highly recommended [53].

Analysis of the microbiological data from the shelf-life study [54] of the final product did not detect pathogen microorganisms. Furthermore, the low counts of spoilage indicator microorganisms (Enterobacteriaceae < 10 CFU/g) evidence both the high efficacy of the applied pasteurization process and strict adherence to hygienic and sanitary measures during packaging and post-processing handling. Mechanistically, the shelf life restriction of these pasteurized matrices suggests an extraordinary biochemical sequence distinct from typical product spoilage models. While other industrial storage trials are very much focused on microbial resurgence prevention, our work showed that optimized *p* value follow-up strongly suppresses pathogen proliferation and severely constrains saprophytic TVC numbers below certain limits up to day 70. LAB constituted the dominant microflora in the final product, exhibiting an exceptionally high correlation (r = 0.993) and nearly identical growth rates (*β*_1_ ≈ 0.025 log/day), which proves that LAB constitute the primary proportion of the TVC. This represents a typical microbial profile for cooked sausages, where microaerophilic conditions inside the packaging preferentially favor the proliferation of LAB [55,56]. Crucially, this progressive LAB proliferation directly drives the primary chemical pathway limiting the shelf life of this pasteurized sausage. As psychrotrophic LAB strains proliferate under cold storage, their endogenous lipolytic enzymes accelerate lipid hydrolytic alterations (acid value), which exhibited a continuous and statistically significant linear growth trend (*R*^2^ = 0.9673), rising from an initial value of 0.42 mg KOH/g to a maximum of 1.26 mg KOH/g (*p* < 0.05) and then slowly declining to 1.19 mg KOH/g at day 70 of shelf life evaluation. This finding suggests a synergistic degradation pathway governed by both endogenous and microbial factors. While psychrotrophic LAB strains established dominance within the microaerophilic packaging environment, their terminal population (3.5 log_10_ CFU/g) remains below conventional threshold limits for standalone microbial spoilage. Consequently, this linear accumulation of free fatty acids is primarily attributed to the joint action of heat-stable, residual endogenous muscle lipases surviving the 71 °C core pasteurization threshold, combined with the metabolic contribution of early-stage extracellular lipolytic enzymes synthesized by the psychrotrophic microbiota. This dual-driven hydrolytic pathway effectively impairs flavor purity before the onset of true microbial structural spoilage or secondary autoxidation.

This continuous growth of free fatty acids, underlined by psychrotrophic LAB endogenous lipolytic enzymes, proves that chemical modifications lead to loss of flavor or aroma before the establishment of true oxidative rancidity or microbial spoilage. The identification of this specific pathway moves the approach from traditional preservation measurement to predictive quality modeling of vacuum-packaged pasteurized emulsions. The average daily increment of free fatty acids was 0.0123 mg KOH/g. This biochemical mechanism indicates that, while the current LAB’s metabolic activity is notably lower than the critical microbiological spoilage threshold (3.5 log CFU/g) at day 70, sufficient free fatty acids are produced that subtly impair the purity of taste and aroma before the development of true oxidative rancidity. After a 70-day storage period, the maximum counts reached 3.5 log CFU/g for LAB and 3.7 log CFU/g for the TVC (Figure 7). These values remain substantially below the critical spoilage threshold for cooked sausages, which is established at 7–8 log CFU/g [57,58].

Throughout the 70-day shelf-life study of the finely minced cooked chicken sausage, the parameters of primary and secondary lipid autoxidation (peroxide value and TBARS value) remained strictly within the optimal technological margins (Figure 8). Lipid oxidation represents a critical quality indicator for food products, as it can directly compromise product safety, shelf-life, sensory attributes, and nutritional value [59,60]. Consequently, numerous analytical techniques have been developed to evaluate the degree of oxidation and the efficacy of incorporated antioxidants [61]. In this study, the peroxide value remained constant at 0.00 mmol/kg over the entire storage period, while the malondialdehyde (MDA) content exhibited a slight increase, reaching a maximum of 0.15 mg MDA/kg on day 70, thereby demonstrating the high oxidative stability of the product. These obtained values are notably lower than those reported in comparable studies [62,63]. Conversely, lipid hydrolytic alterations (acid value) exhibited a continuous and statistically significant linear growth trend (*R*^2^ = 0.9673) rising from an initial value of 0.42 mg KOH/g to a maximum of 1.26 mg KOH/g (*p* < 0.05). The average daily increment of free fatty acids was 0.0123 mg KOH/g, indicating that enzymatically or chemically driven lipid hydrolysis represents the primary chemical pathway limiting the shelf life of this type of finely minced cooked chicken sausage. TBA (Thiobarbituric Acid) Value: The TBA values increased slightly from 0.00 to 0.15 mg MDA/kg. Since values below 0.5 are generally considered “fresh” for meat products, these results confirm that no significant secondary oxidation or rancidity occurred during the 70-day shelf life.

Microbiological stability was confirmed, as no foodborne pathogens were detected at any sampling interval throughout the 70-day storage period. Concurrently, a robust positive correlation (r = 0.993, *p* < 0.001) was observed between TVC and LAB, with final populations reaching safe levels of 3.7 log10 CFU/g and 3.5 log10 CFU/g, respectively. Regarding lipid stability, primary oxidative degradation was completely suppressed, as evidenced by a constant peroxide value of 0.00 mmol/kg from day 1 to day 70. Secondary lipid oxidation remained minimal, with malondialdehyde (MDA) content increasing marginally to 0.15 mg MDA/kg at the end of the study. Conversely, systematic lipid hydrolysis occurred, with the acid value significantly rising from an initial 0.42 mg KOH/g to a maximum of 1.19 mg KOH/g on day 70, establishing enzymatic and chemical hydrolysis as the primary pathway limiting the sensory shelf life of the product.

The changes in the sensory attributes (appearance, color, odor, taste, and consistency) of the final product during the 70-day shelf-life study are presented in Figure 9. Scientific knowledge and technological innovation are pivotal in enabling industries to meet evolving consumer expectations [64]. Contemporary consumers exhibit a heightened interest in the health, safety, and sensory excellence of food products [65,66]. Descriptive sensory analysis, conducted by a trained panel of five experts, revealed two distinct kinetic phases regarding product stability. During the first phase (from day 1 to day 55), the product exhibited exceptional organoleptic stability, with mean scores consistently falling within the ‘exceptionally to highly acceptable’ category (≥3.5 to 5.0). Every descriptive criterion presented in the evaluation matrix, especially in rating systems of scores from 2.0 and 3.0, corresponds to the operational definitions given in reference documentation. The small decrease in sensory scores between “highly acceptable” and “acceptable” at the close of the 70 days’ storage was mainly due to free fatty acid accumulation and lipid hydrolysis, alongside the metabolic activity of LAB. These biochemical changes subtly compromised the purity of the product’s flavor and aroma profile prior to the eventual appearance of oxidative rancidity. This elevated initial quality retention is a direct consequence of the efficacy of the applied commercial pasteurization, governed by the *p* value concept, as well as the structural integrity of the polyamide casing, which successfully prevented moisture loss, surface desiccation, and ambient microbial contamination. In contrast, during the second phase (from day 60 to day 70 of the shelf-life study), a progressive and uniform decline in scores was observed across all evaluated parameters, with mean values on the final day of storage converging within a narrow range between 2.8 and 3.1. This downward trend strongly correlates with concurrent biochemical alterations in the lipid fraction of the product. The release of free fatty acids induced by lipolytic processes is recognized in the literature as a cause of subtle impairments in odor and taste purity prior to the development of true oxidative rancidity. In the present study, severe oxidative rancidity was absent, as evidenced by TBARS values remaining below 0.15 mg MDA/kg, which fully explains the slight decline in scores for these two attributes. Furthermore, the slow metabolic activity of the autochthonous microflora—specifically psychrotrophic LAB contributed to the minor decrease in freshness and consistency observed at the end of the shelf life. Most importantly for product stability, all sensory attributes rated by the five-expert panel remained significantly above the predefined critical limit of acceptability (score 2.0) up to the final 70th day of testing. This demonstrates that no undesirable off-odors, discoloration, or degradation of the sausage batter structure occurred throughout the entire shelf-life period. The sensory results align with the microbiological data; while the product is safe to consume at day 70, the “sensory shelf life” is nearing its end as the product reaches the lower threshold of consumer acceptance.

The longitudinal variations in water activity (*a*_w_) and pH values of wide-diameter finely minced cooked chicken sausages packed in polyamide casing throughout the 70-day cold-chain storage period (0–4 °C) are compiled in Table 5. The initial baseline pH of the raw sausage batter was characterized by an elevated value of 6.21 ± 0.03, primarily driven by the addition of alkaline phosphates and the inclusion of mechanically deboned chicken meat and pea protein isolates, which naturally possess a high buffering capacity. Post-pasteurization (Day 1), the pH of the stabilized gel matrix was measured at 6.18 ± 0.02. Over the course of the 70-day storage, a continuous, statistically significant linear decline (*p* < 0.05) in pH was observed, terminating at a minimum of 5.94 ± 0.03 on the final day. This gradual acidification is mechanistically linked to the progressive metabolic activity of the psychrotrophic LAB community, which exponentially expanded to 3.5 log_10_ CFU/g by day 70. The accumulation of microbial organic acids (mainly lactic acid) within the microaerophilic polyamide casing matrix induced this matrix shrinkage, which strongly correlates with the flat, subtly acidic sensory notes reported by the expert panel during the final phase of the shelf-life trial (days 60–70). Regarding the moisture retention dynamics, the water activity (*a*_w_) maintained exceptional kinetic stability, maintaining an optimum high-moisture environment (0.963–0.965) across the entire shelf-life lifecycle. No statistically significant variations (*p* > 0.05) in *a*_w_ were detected between individual sampling days. This profound stability is a direct consequence of two synchronized parameters: (1) the incorporation of potent hydrocolloids and thickeners (rice flour and pea protein isolates) within the product formulation that strongly immobilized free water molecules within the pre-gel matrix, and (2) the structural integrity of the high-barrier polyamide casings. The impermeable nature of the polyamide polymer effectively suppressed moisture migration, cross-casing desiccation, and weight loss, thereby locking the thermodynamic state of water within the emulsified meat mass. In comparison with established literature data [7], it is evident that these specific intrinsic parameters (*a*_w_ and pH) did not function as an effective antimicrobial hurdle to suppress the growth and proliferation of the residual microbiota. Consequently, for this class of emulsion-type meat products packaged in impermeable polyamide casings, the thermal lethality intensity delivered during pasteurization serves as the primary hurdle governing microbial inactivation. The efficacy of this hurdle is strictly contingent upon two critical extrinsic parameters: the absolute maintenance of casing structural integrity to prevent post-process recontamination, and the continuous, uncompromised preservation of the cold-chain distribution network.

While this paper can offer useful observations for process validation, several limitations have to be considered. First, while the validation studies were conducted with a particular forced-convection thermal processing unit (Maurer chamber) at only one commercial facility, the basic thermodynamic results are based on universal heat transfer concepts. Alternative chamber configurations featuring different airflow dynamics, loading densities, or steam injection geometries will alter the external convective boundary heat transfer coefficient (*h*). While a lower *h* delays the initial warm-up phase, the subsequent internal temperature distribution and cooling inertia within a large-caliber matrix remain fundamentally dictated by internal thermal conduction rather than equipment geometry. The main temperature profiles and subsequent *p* value accumulation are predominantly attributed to the thermal diffusivity of the matrix and the boundary convective heat transfer coefficients rather than proprietary geometry of the equipment. Thus, even though the transient warm-up steps of different equipment configurations may vary, the cooling inertia and matrix-controlled conduction remain fundamentally scalable to any industrial pasteurization system operating under equivalent thermodynamic boundary conditions. Second, the experimental setup was limited to one product matrix (finely minced chicken sausage with a fixed 90 mm diameter size in polyamide casings) but differing compositions in meat emulsions (fat to lean or functional ingredients being present might modify the heat transfer rate and ultimately accumulation of *p* value). Casing diameter scales non-linearly with the surface-area-to-volume ratio, profoundly altering thermal kinetics. In small-caliber matrices (e.g., <40 mm), rapid heat dissipation abruptly halts *p* value accumulation upon cooling phase initiation. Conversely, expanding the caliber increases the thermal core distance, meaning that wider casing diameters exponentially amplify the magnitude and duration of cooling inertia, necessitating customized dynamic monitoring. Changes in the composition of the meat emulsion in the vicinity will affect heat transfer rates. Compared to lipids (k ≈ 0.13 W/m × K), water exhibits far greater thermal conductivity (k ≈ 0.55 W/m × K) and specific heat. Thus, formulations with high moisture content show an increased initial thermal diffusivity, whereas a higher fat fraction decreases heating kinetics, extending core heat retention duration during the water-spraying process. These coupled thermodynamic parameters highlight the need for case-specific empirical validation when modifying product matrices or packaging types.

Furthermore, the 70-day shelf-life assessment was done under the same optimal cold chain refrigeration condition, not simulating actual temperature abuse conditions usually experienced in retail distribution and storage. Lastly, although LAB were the predominant spoilage microflora, their characterization was limited to total enumeration and not species-level molecular identity, so it is not clear which particular strain is driving lipid hydrolysis.

According to the present results, future investigation should mainly concentrate on technological process optimization by systematically decreasing the temperature-holding phase in the chamber, as the achieved cumulative *p* values (76.87–89.56 min) generally exceed the safety limit (*p* ≥ 40 min). This further underscores the importance of this study as an investigation into energy-conserving thermal kinetics in large-diameter matrices. Further optimization steps are necessary to minimize energy consumption and enhance sensory properties without compromising food safety. The main pathway preventing sensory shelf life of the product was identified as either enzymatically or chemically catalyzed lipid hydrolysis. Future work should take into consideration usage of natural antioxidants or antimicrobial extracts of plants to counter lipolytic activities during prolonged storage. Moreover, the development of predictive mathematical and thermodynamic models for simulating heat transfer with respect to different casing diameters (60 mm to 120 mm) subjected to cooling inertia would enable the automation of thermal process termination in commercial applications. Finally, subsequent research will utilize next-generation sequencing (NGS) and metagenomic characterization to precisely map the autochthonous LAB types that contribute to spoilage in large-diameter cooked chicken matrices.

In this study, a key point that stands out is the methodological paradigm shift in validating commercial pasteurization. The cumulative integrated lethality (*p* value) determination can provide an integrated way to see the total thermal energy transferred to meat products, especially those characterized by large calibers. Such a thorough evaluation cannot be achieved by just observing the core temperature at the thermal center of the product. Standard temperature-based monitoring is usually based on the assumption that the thermal process is stopped as soon as the temperature reaches an established critical level and does not address the significant, additional lethality generated by the subsequent cooling process due to thermal inertia. This composite framework of integrated lethality is easily applied to other categories of thermally processed meat formulations, namely coarsely comminuted cooked sausages and smoked meat products. Driving the objective for this research was the operational challenges of verifying excessively high core temperature levels (e.g., 74 °C), often codified as critical limits in the food safety management system of FBOs. To reach the higher core temperatures, meat matrixes are being exposed to sustained thermal stress that is intensely energy demanding and can lead to a devastating compromise of sensory qualities and structural integrity of the finished product. As a result, pasteurization validation through *p* value quantification requires an individualized case-specific methodology. The protocol should adequately incorporate the operational parameters specific to each FBO, such as facility sanitary-veterinary design, thermal processing chamber kinetics, layout configuration, and cooling infrastructure efficiency (air versus water chilling), as well as product-specific intrinsic factors, matrix composition, casing permeability and product caliber.

After validation of commercial pasteurization using *p*-values, the FBO must find the critical point in the processing chamber suitable for routine daily monitoring. Critical temperature thresholds and corresponding *p*-values are then to be continuously monitored at this location during each thermal cycle. A formal validation report of commercial pasteurization shall be produced at least annually by an external expert panel, or more frequently if triggered by specific process modifications, e.g., alterations in the thermal processing profile, commission of a new chamber, or adjustments to product shelf life. In contrast, internal verification must be executed during every operational cycle at the predetermined point within the product core. Additionally, each subsequent comprehensive validation conducted by the expert team lays a solid foundation for further process optimization within the production line.

## 5. Conclusions

In this work, a new scalable validation framework is proposed for the thermal pasteurization of high-caliber (~90 mm) emulsified chicken matrices, and the process-control approach shifts from rigid, temperature-only endpoints towards dynamic, integrated lethality data tracking based on the pasteurization (*p*) value approach. The experimental findings show that closing down the active thermal processing process at a lower core threshold of 71 °C leads to an important thermodynamic cooling inertia. In mass transfer-restricted food, the post-heating period remains the main kinetic driver of microbial inactivation and produces cumulative core *p* values with a value of 76.87 to 89.56 min. This significant thermal build-up is not only significant but also systematically exceeds the stipulated safety buffer (*p* ≥ 40 min) at each monitored vertical direction of the forced-convection thermal chamber, demonstrating that there is no marginal cold point at this temperature domain, and all systemic atmospheric flow occurs within spatial thermodynamic homogeneity. In addition to the regular compliance check step, the increased stability and preservation of this processed product as ready-to-eat evidence the high performance of this well-optimized multi-hurdle preservation system. The combination of targeted integrated lethality and a controlled cold-chain regime (0–4 °C) significantly reduces the prevalence of foodborne pathogens. Throughout the 70-day evaluation period, *Listeria monocytogenes* and *Salmonella* spp. remained below detectable limits, supporting the microbial safety of the product. Autochthonous spoilage microflora, dominant psychrotrophic LAB and TVC, displayed a slow linear proliferation, with maximal populations by day 70 being largely confined within tight microbial spoilage limits. In lipokinetics, basal amounts of peroxide and marginal amounts of malondialdehyde showed absolute suppression in primary and secondary autoxidation pathways. In contrast, discrete enzymatic and chemical hydrolysis of lipid was considered the pivotal biochemical pathway restricting sensory life of the final product. However, by using QDA by a trained expert panel, it was found that all organoleptic attributes significantly surpassed the critical threshold of market acceptability (score 2.0) at all stages until the final stage of storage. From a sectorial/enterprise/financial/environmental perspective, this research builds beyond process verification at-site to provide a translatable thermodynamics framework for advanced food emulsions. This methodology avoids excessive heat handling by mathematically demonstrating that typical fixed temperature validation models (i.e., core heating to 74 °C) can be safely supplanted with dynamic kinetic monitoring. As a result, by fundamentally improving energy efficiency, it retains core nutritional profiles and sensory properties, reduces casing distortion and reduces the carbon footprint of commercial manufacturing facilities. On the whole, this work establishes a solid theoretical background for subsequent industrial implementations, in which predictive mathematical and thermodynamic algorithms for thermally automated process termination at different casing diameters can be integrated with next-generation metagenomic mapping, enabling automated, high-precision quality and safety control for complex food production systems.

## Figures and Tables

**Table 1:** Numerical-descriptive scale for the sensory evaluation of the finished product.

Score	Appearance	Color	Odor	Flavor	Consistency
Extremely acceptable (5)	Casing perfectly smooth, tight, and wrinkle-free; no free water/gelatin release under the casing. Cross-section is perfectly homogeneous, compact, and devoid of air pockets.	Characteristic, uniform, and appealing light-pink color typical of cooked chicken meat; identical color distribution at the periphery and the geometric core.	Extremely pleasant, clean, and typical aroma of cooked chicken meat and mild spices; absolute absence of any off-odors or rancid notes.	Harmonious, rich, and mild flavor of cooked chicken meat with a well-balanced salt-spice profile; leaves a clean, pleasant, and lingering aftertaste.	Firm, pliant, and elastic; slices do not crumble upon cutting, and the product rapidly regains its initial shape under mild finger pressure.
Highly acceptable (4)	Casing is stable with negligible wrinkling at the ends. Cross-section is compact, showing only one or two microscopic air pockets (<1 mm) across the surface.	Typical pink color but with a barely perceptible, minimal shading difference between the outer layer and the core (due to packaging).	Pleasant and recognizable cooked meat odor, with a slightly more pronounced note of the applied spices; no signs of spoilage or rancidity.	Very good flavor with properly balanced saltiness; meat aroma is distinct, though the lingering aftertaste is slightly shorter than Score 5.	Good firmness and satisfactory elasticity; products are juicy, showing no signs of dryness or a rubbery texture during chewing.
Acceptable (3)	Casing shows slight loosening but without deformation of shape. Small, isolated pores are visible on the cross-section, without fat or fluid separation.	Moderately pink to pale color, with slight, localized variations in shade across the cross-sectional surface.	Reduced intensity of the typical meat odor (neutral profile); spice notes dominate, but the profile remains completely clean and free of foreign odors.	Average flavor dominated by added spices or salt, while the natural chicken aroma is less pronounced; free from explicit off-flavors.	Slightly softer consistency, but the product retains its structural integrity; slices can be cleanly and precisely cut into thin pieces.
Marginally acceptable (2)	Casing is visibly loose and wrinkled; noticeable traces of moisture underneath. Cross-section displays a spongier texture with more pores/bubbles.	Distinctly pale, greyish, or with faint grey-yellowish taints at the periphery due to the onset of lipid hydrolysis.	Typical meat odor has faded; a stale, faintly sour, or faint odor is present, indicating initial hydrolytic alterations.	Flavor is flat and non-specific, with slight notes of acidity or excessive saltiness; flavor purity is impaired by free fatty acids.	Consistency is soft, pasty, or mealy; sausage provides low resistance to bite, and slices partially tear during cutting.
Unacceptable (1)	Casing is completely loose, distorted, and sticky; explicit separation of gelatinous mass or fluid. Cross-sectional structure is crumbly and disintegrated.	Distinctly grey, greenish, or yellowish color across the entire cross-section, indicating severe oxidation and microbial spoilage.	Unpleasant, sharp, sour, or rancid odor; clear signs of spoilage are detectable due to high concentrations of lactic acid.	Rancid, sour, bitter, or soapy flavor completely dominates; causes immediate aversion and is entirely unacceptable for consumption.	Extremely soft, mushy, or conversely, completely dry and tough texture; product crumbles upon touch or when attempting to slice.

**Table 2:** Baseline microbial load of the meat batter prior to thermal processing.

Microorganisms (CFU/g ± SD)	Measurement I	Measurement II	Measurement III	*p*-Value
Total viable count	7.02 ± 0.58 ^a^	6.95 ± 0.81 ^a^	7.15 ± 0.74 ^a^	0.905
*Escherichia coli*	2.45 ± 0.91 ^a^	2.37 ± 0.85 ^a^	2.53 ± 0.95 ^a^	0.962
*Enterobacteriaceae*	2.97 ± 0.24 ^a^	3.01 ± 0.36 ^a^	2.82 ± 0.54 ^a^	0.736
*Clostridium* spp.	ND	ND	ND	-
*Bacillus cereus*	ND	ND	ND	-
*Listeria monocytogenes*	ND	ND	ND	-
*Salmonella* spp.	ND	ND	ND	-

**Note**: Values within the same row followed by the same superscript letter (^a^) do not differ significantly (*p* > 0.05) according to one-way ANOVA; SD = Standard Deviation; ND = Not Detected (absence confirmed by the applied method).

**Table 3:** *p* values (minutes) in the product thermal center at the end of the active heating phase/and at the conclusion of measurements.

Thermocouple Probes	Measurement I: Active Heating (min)	Measurement I: Cumulative (min)	Measurement II: Active Heating (min)	Measurement II: Cumulative (min)	Measurement III: Active Heating (min)	Measurement III: Cumulative (min)
Probe 1	20.86	78.06	22.25	78.03	30.39	76.87
Probe 2	29.01	94.91	23.17	82.00	29.25	80.35
Probe 3	33.33	88.83	25.01	84.75	29.2	89.56

**Note**: The values are presented as *p* values at the end of the active heating phase and cumulative *p* values at the conclusion of measurements. The required safety limit for *p* value is > 40 min.

**Table 4:** Comparative analysis of the differences in achieved mean *p* values at the end of the active heating phase and at the conclusion of measurements.

Measurement Cycle	End of Active Heating Phase (min)	Conclusion of Measurement (min)	Absolute Increase (ΔP, min)	Relative Increase (%)
Measurement 1	27.73 ± 2.45 ^a^	87.27 ± 6.12 ^a^	+59.53	+214.7
Measurement 2	23.48 ± 3.11 ^a^	81.59 ± 5.84 ^a^	+58.12	+247.5
Measurement 3	29.61 ± 2.18 ^a^	82.26 ± 4.92 ^a^	+52.65	+177.8
Total mean	26.94 ± 3.14	83.71 ± 3.1	+56.77 ± 3.63	+216.2 ± 34.87

**Note:** Values within the same column followed by the same superscript letter (^a^) do not differ significantly (*p* > 0.05) according to statistical analysis; Absolute Increase (ΔP) in *p* value (minutes) and percentage Increase in *p* value (%). Total mean is presented with standard deviation (±SD).

**Table 5:** Longitudinal tracking of water activity (*a*_w_) and pH values.

Storage Interval (Days)	pH Value (n = 5)	Water Activity (*a*_w_) (n = 5)
Day 1	6.18 ± 0.02 ^a^	0.965 ± 0.002 ^a^
Day 25	6.15 ± 0.03 ^ab^	0.965 ± 0.001 ^a^
Day 40	6.11 ± 0.02 ^b^	0.964 ± 0.002 ^a^
Day 50	6.07 ± 0.04 ^bc^	0.964 ± 0.003 ^a^
Day 55	6.04 ± 0.03 ^cd^	0.963 ± 0.002 ^a^
Day 60	6.01 ± 0.02 ^d^	0.964 ± 0.001 ^a^
Day 65	5.97 ± 0.03 ^de^	0.963 ± 0.002 ^a^
Day 70	5.94 ± 0.03 ^e^	0.963 ± 0.002 ^a^
*p*-value	<0.05	<0.05

**Note:** Data are expressed as Mean Value ± Standard Deviation (SD). Means within the same column sharing the same superscript letter (^a–e^) do not differ significantly (*p* > 0.05) according to a one-way ANOVA followed by Tukey’s post hoc test.

## Data Availability

The original contributions presented in this study are included in the article. Further inquiries can be directed to the corresponding author.

## Supplementary Materials

The following supporting information can be downloaded at: https://www.mdpi.com/article/10.3390/foods15152710/s1, Table S1: First measurement of temperature and *p* values in thermal center of the product and temperature in the chamber medium; Table S2: Second measurement of temperature and *p* values in thermal center of the product and temperature in the chamber medium; Table S3: Third measurement of temperature and *p* values in thermal center of the product and temperature in the chamber medium.

## Abbreviations

The following abbreviations are used in this manuscript:

CCP	Critical Control Point
FBO	Food Business Operator
FFAs	Free Fatty Acids
HACCP	Hazard Analysis and Critical Control Point
LAB	Lactic Acid Bacteria
TVC	Total Viable Count
QDA	Quantitative Descriptive Analysis
